# Quercetin Induces Mitochondrial Apoptosis and Downregulates Ganglioside GD3 Expression in Melanoma Cells

**DOI:** 10.3390/ijms25105146

**Published:** 2024-05-09

**Authors:** Sang Young Seo, Won Seok Ju, Kyongtae Kim, Juhwan Kim, Jin Ok Yu, Jae-Sung Ryu, Ji-Su Kim, Hyun-A Lee, Deog-Bon Koo, Young-Kug Choo

**Affiliations:** 1 Department of Biological Science, College of Natural Sciences, Wonkwang University, Iksan 54538, Jeonbuk, Republic of Korea; tjtkddud463@naver.com (S.Y.S.); jws7895@korea.kr (W.S.J.); alexkim@regreenbio.com (K.K.); moducompany0427@naver.com (J.K.); yjo9703@naver.com (J.O.Y.); 2 Division of Animal Diseases & Health, National Institute of Animal Science, Rural Development Administration, 1500 Kongjwipatjwi-ro, Iseo-myeon, Wanju-gun 55365, Jeonbuk, Republic of Korea; 3 Animal Biotechnology Division, National Institute of Animal Science, Rural Development Administration, 1500 Kongjwipatjwi-ro, Iseo-myeon, Wanju-gun 55365, Jeonbuk, Republic of Korea; 4 Division of Biodrug Evaluation, New Drug Development Center, Osong Medical Innovation Foundation (K-Bio Health), Cheongju 28160, Chungbuk, Republic of Korea; jsryu@kbiohealth.kr; 5 Primate Resources Center (PRC), Korea Research Institute of Bioscience and Biotechnology (KRIBB), Jeongeup 56216, Jeonbuk, Republic of Korea; kimjs@kribb.re.kr; 6 Center for Animal Resources Development, Wonkwang University, Iksan 54538, Jeonbuk, Republic of Korea; leeha85@wku.ac.kr; 7 Department of Biotechnology, College of Engineering, Daegu University, Gyeongsan 38453, Gyeongbuk, Republic of Korea; dbkoo@daegu.ac.kr; 8 Institute for Glycoscience, Wonkwang University, Iksan 54538, Jeonbuk, Republic of Korea

**Keywords:** apoptosis, cell-cycle arrest, ganglioside, knockdown, malignant melanoma, quercetin

## Abstract

Malignant melanoma represents a form of skin cancer characterized by a bleak prognosis and heightened resistance to traditional therapies. Quercetin has demonstrated notable anti-carcinogenic, anti-inflammatory, anti-oxidant, and pharmacological effects across various cancer types. However, the intricate relationship between quercetin’s anti-cancer properties and ganglioside expression in melanoma remains incompletely understood. In this study, quercetin manifests specific anti-proliferative, anti-migratory, and cell-cycle arrest effects, inducing mitochondrial dysfunction and apoptosis in two melanoma cancer cell lines. This positions quercetin as a promising candidate for treating malignant melanoma. Moreover, our investigation indicates that quercetin significantly reduces the expression levels of ganglioside GD3 and its synthetic enzyme. Notably, this reduction is achieved through the inhibition of the FAK/paxillin/Akt signaling pathway, which plays a crucial role in cancer development. Taken together, our findings suggest that quercetin may be a potent anti-cancer drug candidate for the treatment of malignant melanoma.

## 1. Introduction

Melanoma, an aggressive form of skin cancer originating from melanocytes [1], poses a significant challenge with its escalating incidence and bleak prognosis upon metastasis [2,3,4,5]. Despite available therapies like molecular-targeted and immunotherapies, treatment efficacy is hampered by drug resistance and suboptimal patient response [1,6,7]. Therefore, comprehending the intricate mechanisms driving melanoma progression is imperative for uncovering potential therapeutic targets.

Quercetin (3,3′,4′,5,7-pentahydroxyflavone), a polyphenolic flavonoid present in various plants and utilized as a dietary supplement, raises interest due to its low-toxicity profile [8]. Intriguingly, several researchers propose its relevance in anti-oxidant, anti-inflammatory, and anti-cancer activities specific to melanoma [9,10]. Additionally, it induces cell-cycle arrest and activates anti-oxidant pathways across diverse cancer types [11,12,13]. While it has demonstrated inhibitory effects on migration and invasion in certain cancers, including skin cancer [14,15,16], the precise mechanism of its anti-cancer action remains elusive. Ongoing efforts persist in the quest for novel melanoma biomarkers to enhance treatment strategies.

Simultaneously, gangliosides, a subtype of glycosphingolipids prevalent on cell surfaces, particularly abundant in the nervous tissues of various animals [17], play a pivotal role in cell functions like proliferation, adhesion, migration, and interactions within the cell microenvironment in different cancers [18,19,20,21]. Expression patterns and the underlying mechanisms of gangliosides in normal and cancer cells have been explored by several researchers [22,23,24]. Notably, human melanomas predominantly feature ganglioside GM3 and GD3 on their cell surfaces [25]. The enhanced expression of ganglioside GD3 has been associated with accelerated cell growth and increased invasiveness in certain cancers, such as small-cell lung cancer [26]. However, a comprehensive understanding of the role of gangliosides in cancers, particularly in relation to the pharmacological actions of quercetin in melanoma, requires further exploration.

Focal adhesion kinase (FAK) emerges as a key player in maintaining cellular architecture by forming molecular complexes with glycolipids, integrins, and itself [27]. Particularly implicated in crucial biological processes like cancer survival, migration, and invasion [28,29], FAK also regulates proliferative activities involving PI3K, Akt, mTOR, Ras, and ERK/MAPK in various cancers [30,31]. Despite its significance, the molecular interactions between ganglioside GD3 and FAK in the regulation of melanoma functions, including tumor initiation and metastasis, remain unexplored.

The intricate relationship between quercetin and the mechanisms involving gangliosides in melanoma cells remains enigmatic to date. To unravel the intricacies of quercetin’s actions, gangliosides, and melanoma cells, we thus investigate the interplay between quercetin and gangliosides, focusing specifically on ganglioside GD3. Our findings suggest quercetin has potential efficacy in melanoma cells and is involved in the synthesis of gangliosides in melanoma.

## 2. Results

### 2.1. Quercetin Decreased Viability and Changed Cellular Morphology in Melanoma Cells

**Figure 1:**
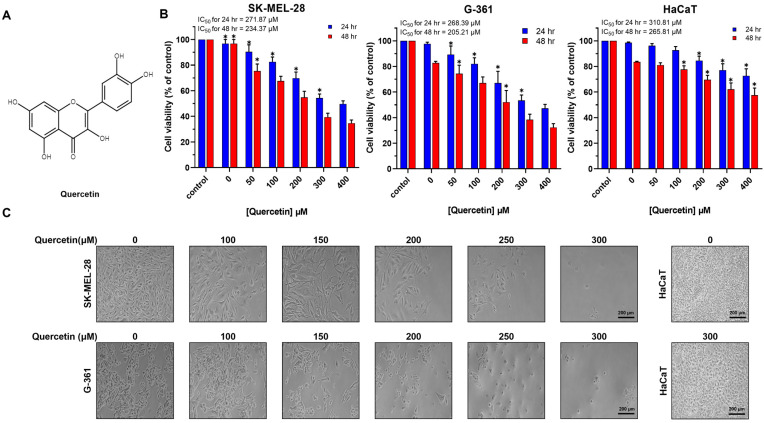
Effects of quercetin on cell viability of malignant melanoma cells. (**A**) Chemical structure of quercetin. (**B**) Cell viability of melanoma SK-MEL-28 and G-361 and non-tumoral HaCaT cells treated with different concentrations of quercetin for 24 and 48 h (hr). Viability was detected using MTT assay kit. (**C**) Morphological changes in quercetin-treated SK-MEL-28, G-361, and HaCaT cells for 24 h and corresponding untreated controls. Data represent mean ± standard deviation (SD) of three independent experiments (*n* = 3, * *p* < 0.05).

To investigate the influence of quercetin on the viability of melanoma cells, SK-MEL-28 and G-361 lines, alongside non-tumorigenic HaCaT epidermal cells, were exposed to a spectrum of quercetin concentrations at two distinct time intervals [24 and 48 h (hr)]. The chemical structure of quercetin used in this study is shown in Figure 1A. Cell viability was subsequently assessed using an MTT assay (Figure 1B). The half-maximal inhibitory concentration (IC_50_), defined as the concentration necessary to reduce cellular viability by 50%, was determined to be 271.87 μM and 268.39 μM for SK-MEL-28 and G-361 cells, respectively. In contrast, the IC_50_ for HaCaT control cells was 310.81 μM (Figure 1B). A pronounced reduction in viability was observed across both melanoma cell lines when exposed to quercetin concentrations ranging from 0 to 400 μM. In contrast, HaCaT cells exhibited no discernable changes in viability even at concentrations as high as 300 μM. To further investigate the cytotoxic effects of quercetin, morphological alterations were evaluated in SK-MEL-28 and G-361 cells following treatment with increasing doses of quercetin (100–300 μM) for 24 h. Quercetin treatment resulted in a marked decrease in cellular number and a concomitant reduction in cell size, accompanied by a transformation toward a more spherical morphology (Figure 1C). Conversely, HaCaT cells exposed to 300 μM quercetin displayed no significant morphological changes.

### 2.2. Quercetin Induced Apoptosis in Melanoma Cells

**Figure 2:**
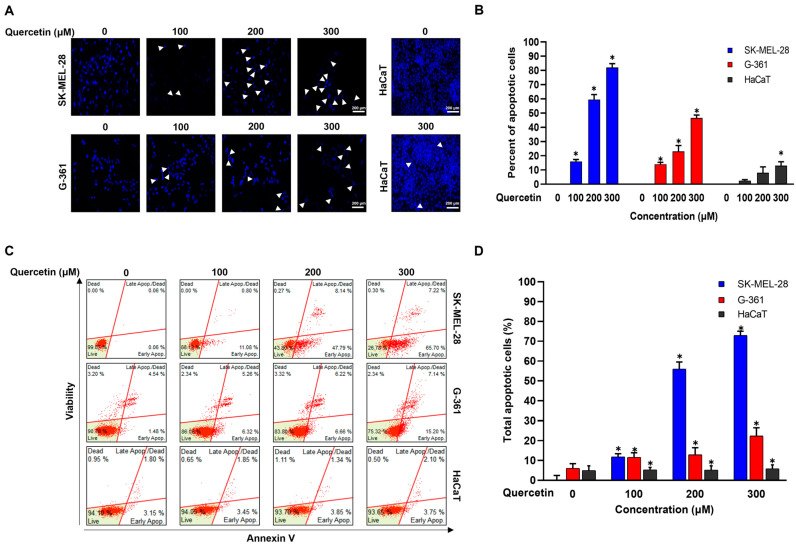
Apoptosis effects induced by quercetin in malignant melanoma cells. SK-MEL-28, G-361, and HaCaT cells were cultured without or with quercetin (100, 200, and 300 µM) for 24 h. (**A**) Representative fluorescence images (magnification: ×200) of DAPI stained cells. White arrows indicate DNA fragmentation and nuclear condensation. (**B**) DNA fragmentation and nuclear condensation were quantified. Data represent mean ± SD (*n* = 3, * *p* < 0.05). (**C**) Quantitative detection of Annexin-V/7-ADD-positive cells using Muse Cell Analyzer. SK-MEL-28, G-361, and HaCaT cells were treated with quercetin (100, 200, and 300 µM) for 24 h. Cells stained with Annexin-V only were defined as early apoptotic. Annexin-V/7-ADD double-stained cells were defined as late apoptotic. In Annexin-V/7-ADD results, lower left quadrant represents live cells, lower right quadrant represents early apoptotic cells, upper right quadrant represents late apoptotic cells, and upper left quadrant represents dead cells. (**D**) Percentage of total apoptotic cell population was quantified. Data are presented as mean ± SD (*n* = 3, * *p* < 0.05).

The effects of quercetin on cellular apoptosis in SK-MEL-28, G-361, and HaCaT cells were determined by nuclear morphology using DAPI staining and Annexin-V/7-ADD staining analysis, and these results were verified by images of the morphological changes. It was confirmed that quercetin (100, 200, and 300 μM for 24 h) induced nuclear condensation and perinuclear apoptotic bodies in melanoma cells in a concentration-dependent manner. Morphological and nuclear changes were also induced in HaCaT cells by quercetin (300 μM), although to a lesser degree (Figure 2A). The percentage of cells with nuclear fragmentation in quercetin-treated cells vs. non-treated cells is shown in Figure 2B. Additionally, the apoptotic effect of quercetin on SK-MEL-28 and G-361 melanoma cells was evaluated after 24 h using the Annexin-V/7-ADD assay (Figure 2C). For the upper panel in Figure 2C, the average percentage of early apoptotic cells in the SK-MEL-28 group was 0.06, 11.08, 47.79, and 65.70% after treatment with 0, 100, 200, and 300 μM quercetin, respectively, and the average ratio of dead cells and late apoptotic cells was 0.06, 0.80, 8.14, and 7.22%, respectively. The proportion of live cells in the control group was 99.87%, which decreased to 88.12, 43.80, and 26.78% as the concentration of quercetin increased. Similarly, the middle panel in Figure 2C shows the proportions in the G-361 group. The average percentage of cells in the early apoptosis phase was 1.48, 6.32, 6.66, and 15.20% after treatment with 0, 100, 200, and 300 μM quercetin, respectively, and the average percentage of dead cells and late apoptotic cells was 4.54, 5.26, 6.22, and 7.14%, respectively. The proportion of live cells in the control group was 90.78, 86.08, 83.80, and 75.32% as the concentration of quercetin increased. The lower panel in Figure 2C shows the proportions in the HaCaT group. The average percentage of cells in the early apoptosis phase was 3.15, 3.45, 3.85, and 3.75% after treatment with 0, 100, 200, and 300 μM quercetin, respectively, and the average percentage of dead cells and late apoptotic cells was 1.80, 1.85, 1.34, and 2.10%, respectively. The proportion of live cells in the control group was 94.10, 94.05, 93.70, and 93.65% as the concentration of quercetin increased. The percentage of the total apoptotic cell population in quercetin-treated cells vs. non-treated cells is shown in Figure 2D.

### 2.3. Quercetin Regulated Cell-Cycle Arrest and Apoptosis-Related Protein in Melanoma Cells

**Figure 3:**
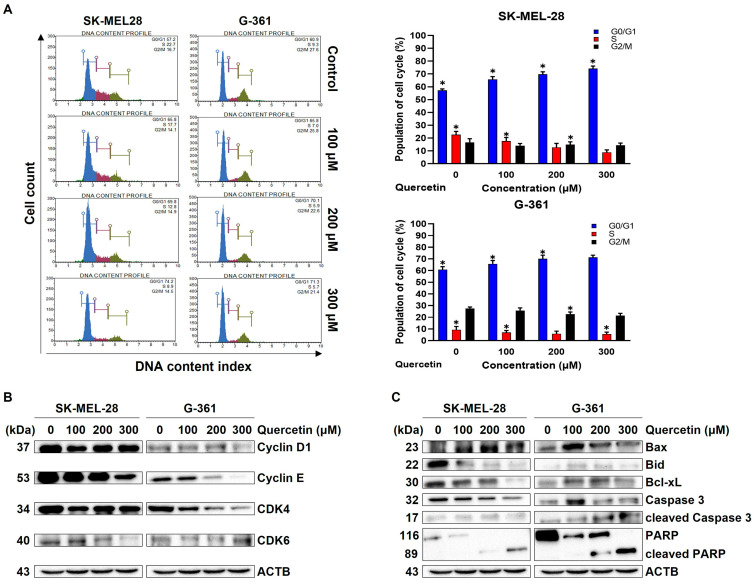
Quercetin regulates cell-cycle G0/G1 arrest and mitochondrial apoptosis in malignant melanoma cells. Malignant melanoma SK-MEL-28 and G-361 cells were cultured without quercetin (control) or with quercetin (100, 200, and 300 µM) for 24 h. (**A**) Malignant melanoma SK-MEL-28 and G-361 cells were washed with DPBS, fixed in 70% ethanol overnight, stained with Muse^TM^ Cell-Cycle reagent, and analyzed for DNA content using Muse^TM^ Cell Analyzer. Percentage of cell-cycle phase was quantified. Data represent mean ± SD (*n* = 3, * *p* < 0.05). (**B**) Dose-dependent effect of quercetin on cell-cycle-related proteins, such as cyclin D, cyclin E, cdk4, and cdk6. Malignant melanoma SK-MEL-28 and G-361 cells were treated with different concentrations of quercetin for 24 h. ACTB was used as loading control. ACTB: β-actin. (**C**) Dose-dependent effect of quercetin on apoptosis-related proteins, such as Bax, Bid, Bcl-xL, caspase 3, cleaved caspase 3, and PARP. Malignant melanoma SK-MEL-28 and G-361 cells were treated with different concentrations of quercetin for 24 h. ACTB was used as loading control. ACTB: β-actin.

To investigate the inhibitory effects of quercetin on cell-cycle arrest in SK-MEL-28 and G-361 cells, both cell lines were exposed to quercetin for 24 h, and the cell-cycle distribution was assessed by FACS analysis (left panel Figure 3A). In SK-MEL-28 cells, the proportions of G0/G1 cells were 57.2%, 65.8%, 69.8%, and 74.2% when treated with 0, 100, 200, and 300 μM quercetin, respectively. In G-361 cells, the percentages of G0/G1 cells were 60.9%, 65.8%, 70.1%, and 71.3% under 0, 100, 200, and 300 μM quercetin treatment, respectively. Thus, quercetin demonstrated a dose-dependent increase in the levels of G0/G1 cells in SK-MEL-28 and G-361, respectively (Figure 3A right panel). To confirm these changes in the cell cycle, the expression levels of G0/G1 phase-related proteins (cyclin D, cyclin E, CDK4, CDK6) were analyzed after 24 h of quercetin treatment. As shown in Figure 3B, the expression levels were diminished in a dose-dependent manner. To delve deeper into the relationship between alterations in the cell cycle and cell apoptosis, apoptosis-related proteins, including Bid, Caspase 3, and Poly ADP-ribose polymerase (PARP), were examined; they underwent significant truncation. Specifically, the expression levels of Bax, cleaved Caspase 3, and cleaved PARP were increased, whereas the expression of the anti-apoptotic protein Bcl-xL decreased, as shown in Figure 3C.

### 2.4. Quercetin Changed MMP and Induced Release of Cytochrome C from Intermembrane Space in Melanoma Cells

**Figure 4:**
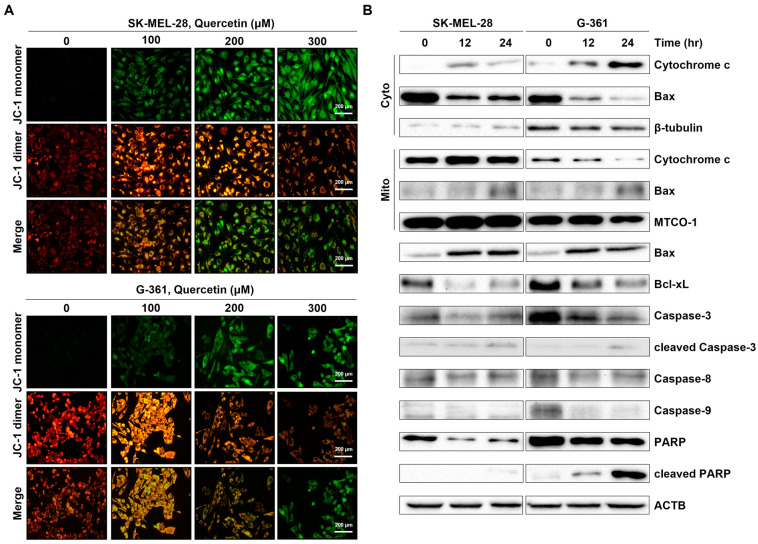
Changes in mitochondrial membrane potential of malignant melanoma cells. (**A**) Representative images of mitochondrial membrane depolarization (JC-1 staining) by immunofluorescence microscopy. Malignant melanoma SK-MEL-28 and G-361 cells were treated with quercetin (100, 200, and 300 μM) for 24 h. Mitochondrial membrane depolarization increased in dose-dependent manner. (**B**) The mitochondrial apoptotic process in malignant melanoma SK-MEL-28 and G-361 cells was determined by immunoblot analysis of mitochondrial, cytosolic, and whole-protein levels. Mitochondrial fraction was confirmed using mitochondrial marker MTCO1, and cytosolic fraction was confirmed using β-tubulin. ACTB was used as loading control in total protein. ACTB: β-actin. All data shown were representative of at least three independent experiments.

To elucidate whether quercetin instigates apoptosis in SK-MEL-28 and G-361 cells through the mitochondrial pathway, we determined mitochondrial membrane potential (MMP) by JC-1 staining and scrutinized Bcl-2 family proteins and cytochrome c levels. As shown in Figure 4A, there was a discernible, dose-dependent augmentation in JC-1 monomers (green fluorescence), signifying MMP depolarization in melanoma cells.

Next, investigations probed the time-dependent expression of mitochondrial and cytosolic fractions of proteins linked to the mitochondrial apoptotic pathway in both cell lines. As depicted in Figure 4B, quercetin treatment prompted a gradual release of mitochondrial cytochrome c into the cytosol of SK-MEL-28 and G-361 cells, coupled with a simultaneous elevation in mitochondrial Bax levels. This was succeeded by a pronounced escalation in cleaved caspase 3 and cleaved PARP, while pro-apoptotic proteins such as Bid and caspase 9, along with the anti-apoptotic protein Bcl-xL, exhibited noteworthy downregulation.

### 2.5. Quercetin Regulated Expression of Proliferation-Related Molecules in Melanoma Cells

**Figure 5:**
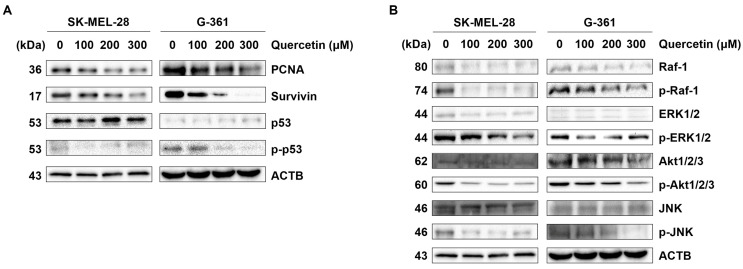
Quercetin suppressed the proliferation of proteins involved in the Raf-Akt pathway in malignant melanoma cells. (**A**) Protein expression levels of PCNA, survivin, p53, and phosphorylated p53 detected using immunoblotting analysis in a dose-dependent panel for 24 h. (**B**) Protein expression levels of Raf-1, ERK1/2, Akt1/2/3, JNK, and phosphorylated Raf-1, ERK1/2, Akt1/2/3, and JNK were detected using immunoblotting analysis in a dose-dependent panel for 24 h. ACTB was used as a loading control. ACTB: β-actin. All data shown were representative of at least three independent experiments.

DNA replication marker PCNA mirrors cell proliferation, while survivin, implicated in tumor growth and spread, plays a key role in these processes. Given the PI3K/Akt pathway’s central role in cell survival and proliferation, its activity was evaluated through immunoblotting following quercetin treatment in melanoma cells. Both survivin and PCNA, established markers of cellular growth and proliferation, displayed a dose-dependent decrease (Figure 5A). Additionally, p-p53 decreased in a concentration-dependent manner relative to total p53.

Melanoma progression is known to involve two main signaling pathways: Ras-Raf-MEK-ERK and Ras-Raf-MAPK/PI3K-Akt. Therefore, the regulation of melanoma progression was closely examined. As shown in Figure 5B, phosphorylated Raf (p-Raf) and phosphorylated Akt (p-Akt) showed a significant reduction in a dose-dependent manner, while phosphorylated ERK did not exhibit a dose-dependent decrease.

### 2.6. Quercetin Inhibited Malignant Melanoma Cell Migration

Tumor metastasis and migration, hallmarks of many cancers, are fundamentally linked to the ability of cancer cells to invade surrounding tissues and distant sites. However, the effect of quercetin on the migratory prowess of melanoma cells remains unclear. To shed light on this, we systematically investigated the impact of quercetin (250 µM) on melanoma cell migration using a wound healing assay.

**Figure 6:**
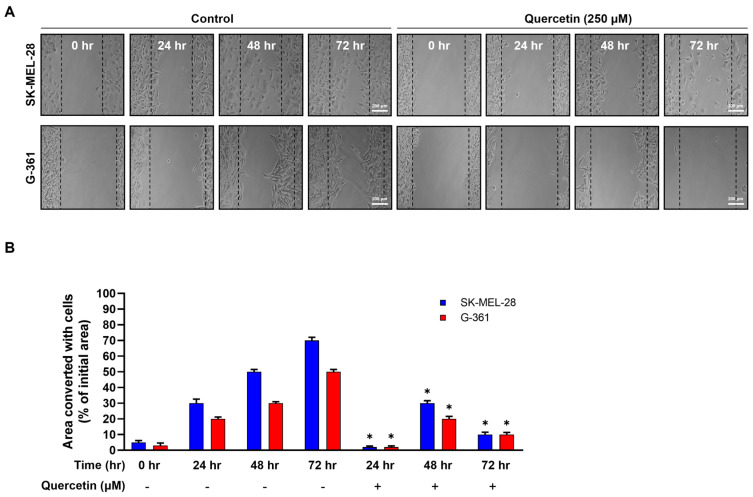
Quercetin significantly inhibited the metastatic ability of malignant melanoma cells. (**A**) Effect of quercetin on the migration of malignant melanoma SK-MEL-28 and G-361 cells. Malignant melanoma SK-MEL-28 and G-361 cells were treated with quercetin (250 μM). Malignant melanoma cells were scratched using sterile pipette tips and incubated with or without (control group) quercetin. Samples were observed under a light microscope at 24, 48, and 72 h. (**B**) The area covered with cells was quantified. The blue square and red square represent SK-MEL-28 and G-361 cells, respectively. Data represent mean ± SD (*n* = 3, * *p* < 0.05).

As shown in Figure 6A, clearly demonstrates that quercetin treatment significantly dampened the migratory capacity of both SK-MEL-28 (upper panel) and G-361 (lower panel) melanoma cells. Specifically, quercetin-treated SK-MEL-28 cells covered only 2.6%, 31.2%, and 13.43% of the denuded area at 24, 48, and 72 h post-scratch, respectively. Similarly, G-361 cells exposed to quercetin exhibited coverage of 2.54%, 20.4%, and 9.5% at the corresponding time points (Figure 6B).

Comparing quercetin-treated melanoma cells with their untreated counterparts revealed a modest increase in the wound closure ratio. Notably, both cell population and migratory capacity decreased over time, suggesting a potential inhibitory effect of quercetin on melanoma cell migration.

### 2.7. Quercetin Reduced Ganglioside GD3 Expression in Melanoma Cells

**Figure 7:**
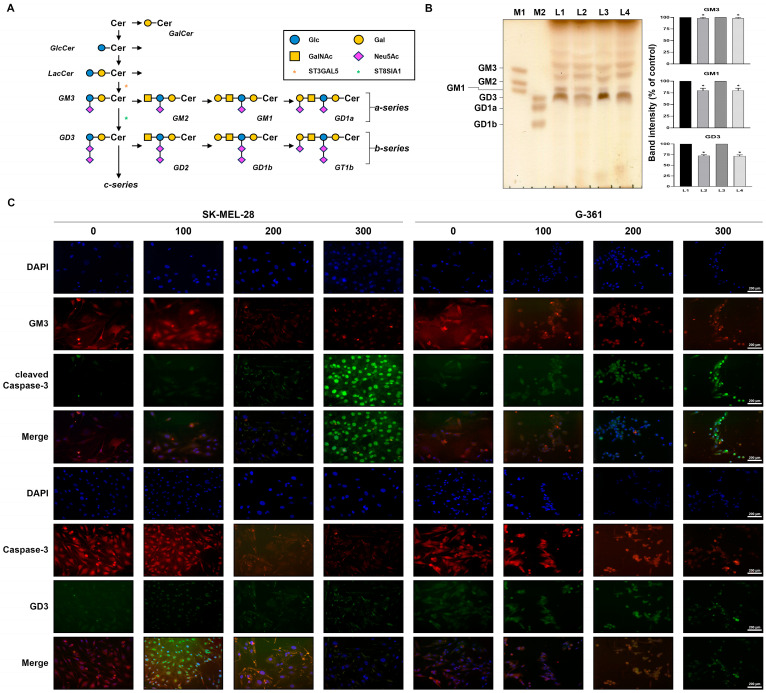
Changes in ganglioside expression in quercetin-treated malignant melanoma cells. (**A**) Common ganglioside biosynthesis. G: ganglioside; M: monosialo; D: disialo; numbers denote carbohydrate sequence. Cer: ceramide; GlcCer: glucosylceramide; LacCer: lactosylceramide; GalNAc: N-acetylgalactosamine; ST3GAL5, ST3 beta-galactoside alpha-2,3-sialyltransferase 5; ST8SIA1, ST8 alpha-N-acetylneuraminide alpha-2,8-sialyltransferase 1. (**B**) HPTLC analysis of ganglioside in untreated SK-MEL-28 cells (L1), SK-MEL-28 cells treated with quercetin (250 μM for 24 h) (L2), untreated G-361 cells (L3), and G-361 cells treated with quercetin (250 μM for 24 h) (L4). M, marker; L, line; M1 and M2, ganglioside standard mixture marker (left panel); quantification of band intensity for GM3, GM1, and GD3 in malignant melanoma SK-MEL-28 and G-361 cells (right panel). Data represent mean ± SD (*n* = 3, * *p* < 0.05). (**C**) Immunofluorescence microscopy analysis of quercetin-treated malignant melanoma SK-MEL-28 and G-361 cells. Malignant melanoma SK-MEL-28 and G-361 cells were treated with different concentrations of quercetin (100, 200, and 300 μM) for 24 h, and cells were immunostained with anti-GM3 or anti-GD3 and anti-cleaved caspase 3. Signals were detected with Alexa fluor 488-conjugated anti-goat and Alexa fluor 647-conjugated anti-mouse secondary antibody. The result shown in (**C**) were representative of at least three independent experiments.

Gangliosides, complex sugar molecules, play critical roles in cancer development, as detailed in Figure 7A. Among various gangliosides, GM3 and GD3 stand out for their involvement in cell growth, movement, invasion, and interactions in different cancer types [32,33]. Therefore, we investigated the potential of quercetin to modulate ganglioside expression in melanoma cells using High-Performance Thin-Layer Chromatography (HPTLC).

As shown in Figure 7B, treating melanoma cells with 250 µM quercetin for 24 h reduced GM3 and GD3 expression. Notably, this effect was not observed in HaCaT cells (Figure S1C). To delve deeper, we examined the impact of quercetin on mRNA and protein levels of ganglioside synthases in melanoma cells treated with 250 µM quercetin for 24 h. This revealed decreased mRNA expression of ST3GAL5 and ST8SIA1 (Figure S1A) and reduced protein levels of GM3 and GD3 synthases (Figure S1B). Interestingly, HaCaT cells showed no significant changes in ganglioside synthesis protein or mRNA levels between control and quercetin-treated groups (Figure S1D,E).

Furthermore, we employed nuclear staining and confocal microscopy to analyze the expression patterns of ganglioside GM3 or GD3 alongside the apoptosis marker, cleaved caspase 3, in melanoma cells (Figure 7C). Using DAPI for nuclear staining and specific antibodies for GD3, GM3, and cleaved caspase 3, we observed a dose-dependent decrease in the expression of all three in quercetin-treated melanoma cells undergoing apoptosis.

### 2.8. Reduced Ganglioside Expression Levels by ST8SIA1 and/or ST3GAL5 Knockdown in Melanoma Cells

**Figure 8:**
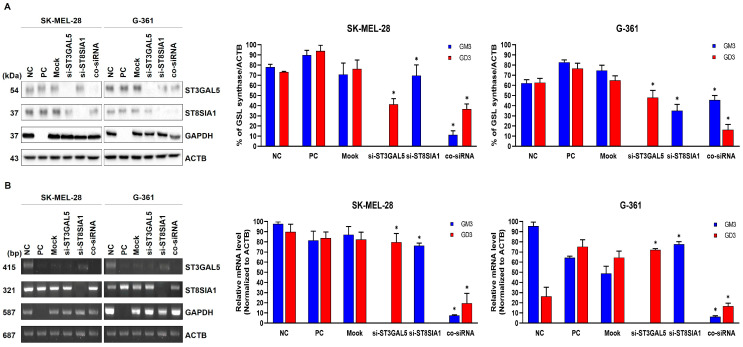
Effects of knockdown of ganglioside GM3 synthase or GD3 synthase siRNA on malignant melanoma cells. (**A**) Knockdown of ganglioside GM3 or GD3 synthase on malignant melanoma SK-MEL-28 and G-361 cells. siRNA against ganglioside GM3 or GD3 synthase was transfected with Lipofectamin^®^3000 reagent, and protein levels of ganglioside GM3 and GD3 synthase were examined after four days of transfection using immunoblotting (left panel). Blue and red squares represent ganglioside GM3 and GD3 synthase protein levels, respectively (right panel). ACTB was used as loading control. ACTB: β-actin. All data are presented as mean percentage levels ± SD (*n* = 3, * *p* < 0.05). (**B**) RT-PCR analysis of melanoma SK-MEL-28 and G-361 cells treated with ganglioside GM3 and GD3 synthase siRNA (left panel). Blue and red squares represent ganglioside GM3 and GD3 synthase protein levels, respectively (right panel). ACTB was used as loading control. ACTB: β-actin. All data are presented as mean percentage levels ± SD (*n* = 3, * *p* < 0.05).

Due to the heightened expression of ganglioside GM3 and ganglioside GD3, along with their respective synthases, in melanoma cells, we embarked on an investigation to elucidate the functional ramifications of these gangliosides in contact inhibition. Employing sophisticated siRNA technology, we orchestrated knockdowns targeting ST3GAL5 and ST8SIA1, resulting in three distinctive knockdown cell phenotypes: knockdown-ST3GAL5 (si-ST3GAL5), knockdown-ST8SIA1 (si-ST8SIA1), and a concurrent knockdown of both ST3GAL5 and ST8SIA1 (co-siRNA). Control cells were transfected with a reference control siRNA. The impact of these knockdowns on protein levels was assessed precisely 24 h post-transfection through the meticulous application of immunoblotting techniques (Figure 8A).

In the SK-MEL-28 cell lines, relative to the control counterparts, ganglioside GM3 synthase protein levels saw reductions of 45.8% in si-ST3GAL5, 11.1% in si-ST8SIA1, and a substantial 90.0% in co-siRNA. Furthermore, ganglioside GD3 synthase protein levels exhibited declines of 54.5% in si-ST3GAL5, 58.5% in si-ST8SIA1, and 49.1% in co-siRNA. Analogously, in the G-361 cells, ganglioside GM3 synthase protein levels experienced reductions of 54% in si-ST3GAL5, 57.7% in si-ST8SIA1, and 48.8% in co-siRNA. Likewise, ganglioside GD3 synthase protein levels demonstrated decreases of 43.8% in si-ST3GAL5, 12.3% in si-ST8SIA1, and a substantial 88.1% in co-siRNA.

The consequential impact of these knockdowns on mRNA levels was meticulously scrutinized 24 h post-transfection using state-of-the-art Reverse Transcription Polymerase Chain Reaction (RT-PCR) analysis (Figure 8B). In SK-MEL-28 cells, relative to the control group, ganglioside ST3GAL5 mRNA levels exhibited marked reductions of 66% in si-ST3GAL5, 89% in si-ST8SIA1, and a remarkable 92% in co-siRNA. Correspondingly, ganglioside ST8SIA1 mRNA levels witnessed reductions of 37% in si-ST3GAL5, 54% in si-ST8SIA1, and 57% in co-siRNA. In the G-361 cells, ganglioside ST3GAL5 mRNA levels underwent significant declines of 66% in si-ST3GAL5, 89% in si-ST8SIA1, and a noteworthy 92% in co-siRNA. Moreover, ganglioside ST8SIA1 mRNA levels exhibited marked reductions of 57% in si-ST3GAL5, 85% in si-ST8SIA1, and 90% in co-siRNA.

### 2.9. Quercetin and Knockdown Ganglioside Decreased Gene Expression of FAK, Paxillin, and Akt Signaling Pathways

**Figure 9:**
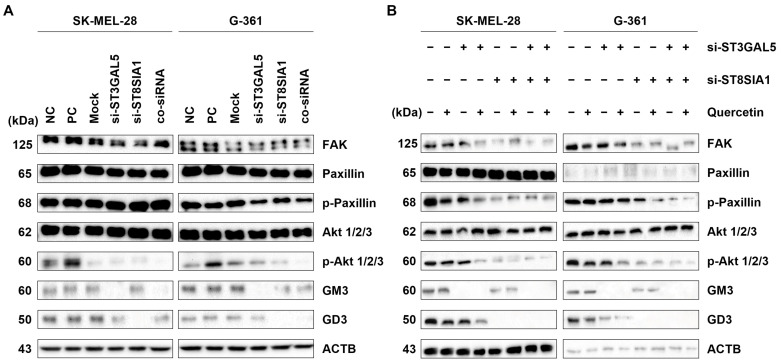
Ganglioside siRNA inhibits relative protein levels of FAK, paxillin, and Akt in malignant melanoma cells. (**A**) Knockdown of ganglioside GM3 or GD3 synthase on malignant melanoma SK-MEL-28 (left panel) and G-361 (right panel) cells. Effects of knockdown of ganglioside GM3 or GD3 synthase on phosphorylation levels of paxillin and Akt in malignant melanoma SK-MEL-28 and G-361 cells. Reduction in tyrosine phosphorylation was observed, and its total protein level was not affected. ACTB was used as loading control. ACTB: β-actin. All data shown were representative of at least three independent experiments. (**B**) Knockdown of ganglioside GM3 or GD3 synthase treated with or without quercetin on malignant melanoma SK-MEL-28 and G-361 cells. Protein levels of FAK, paxillin, and Akt and phosphorylation levels of Akt and paxillin were examined after 24 h in untreated and quercetin-treated (250 µM) panel cells using immunoblotting analysis. ACTB was used as loading control. ACTB: β-actin. All data shown were representative of at least three independent experiments.

p130Cas and paxillin orchestrate cellular proliferation and invasion in melanoma cells [34]. We compared the proliferation dynamics of melanoma cells transfected with ganglioside siRNA to untransfected controls. To elucidate the key proteins driving the observed increase in proliferation and invasion, we meticulously performed immunoblotting on cell lysates after ganglioside siRNA transfection (Figure 9A). Both single and double ganglioside siRNA transfections exhibited a discernible upregulation of focal adhesion kinase (FAK) and phosphorylated paxillin (p-paxillin) while concurrently downregulating paxillin, phosphorylated Akt (p-Akt), GM3 synthase, and GD3 synthase.

To explore the interplay between quercetin and ganglioside siRNA-transfected melanoma cells, we conducted another immunoblotting analysis (Figure 9B). Melanoma cells transfected with either single or double ganglioside siRNA were treated with 250 µM quercetin for 24 h. Both untreated and treated cells underwent immunoblotting. Regardless of ganglioside siRNA presence, both quercetin-treated and untreated melanoma cells showed consistent decreases in FAK, paxillin, and p-Akt protein levels. However, a comparative analysis revealed a more pronounced reduction in protein levels with ST8SIA1 siRNA and quercetin treatment compared to ST3GAL5 siRNA and quercetin treatment. Additionally, double siRNA groups displayed a more substantial reduction in FAK, p-paxillin, and p-Akt protein levels compared to single siRNA groups.

## 3. Discussion

In the era of targeted cancer therapies, the development of drugs tailored to specific cancer types has revolutionized cancer treatment [1,6]. Despite significant progress, a subset of melanoma patients faces challenges due to the emergence of drug resistance [35,36]. To enhance malignant melanoma treatment, it is crucial to identify biomarkers, refine early detection methods, and discover new targets for molecular-targeted therapies.

Quercetin, a naturally occurring compound renowned for its diverse health benefits, has undergone extensive investigation for its potent anti-cancer properties [37,38,39]. In the context of melanoma, our study aimed to assess quercetin’s potential as an anti-melanoma agent. Initial investigations revealed a substantial reduction in cell viability and the induction of apoptosis in malignant melanoma cells (SK-MEL-28 and G-361 cell lines) treated with quercetin. Importantly, the impact on human normal epithelial cells (HaCaT cells) was less pronounced. To maintain methodological consistency, we limited the quercetin concentration for melanoma cell treatment to a maximum of 250 µM within a 24 h timeframe, considering modest toxicity in HaCaT cells at 300 µM after 48 h. This approach aligns with the careful consideration required for optimal treatment concentrations in the dynamic genetic landscape inherent to melanoma, characterized by intra-tumor heterogeneity driven by sporadic genetic alterations [40,41,42].

Quercetin’s effect on cell-cycle regulation was explored through FACS analysis, revealing a significant induction of G0/G1 cell-cycle arrest in melanoma cells, consistent with previous research [13,43]. This underscores quercetin’s potential to impede the growth of malignant melanoma cells by modulating cell-cycle checkpoints.

Alterations in mitochondrial membrane potential and changes in mitochondrial-apoptosis-pathway-related proteins further emphasized quercetin’s anti-melanoma efficacy, aligning with previous studies [44,45,46]. The considerable reduction in the recovery ability and mobility of melanoma cells following quercetin treatment further supports its anti-melanoma properties [15].

The examination of ganglioside expression in melanoma cells revealed elevated levels of both ganglioside GD3 and GM3, consistent with prior reports [47,48]. Quercetin treatment effectively decreased the expression levels of these gangliosides, aligning with findings using other flavonoid drugs [49]. The data suggest ganglioside GM3 and GD3 as potential specific markers for melanoma cells, potentially serving as therapeutic targets in these cancers.

Knockdown experiments targeting gangliosides GM3 and GD3 resulted in reduced cell growth and migration in melanoma cells, accompanied by decreased expression of FAK [50,51] and paxillin [52,53]. Interestingly, GD3-knockdown cells exhibited weaker expression of these proteins compared to GM3-knockdown cells, suggesting that GD3 expression may be more closely associated with malignant characteristics, such as cell growth and migration.

The combined knockdown of gangliosides GM3 and GD3 led to more pronounced inactivation of FAK and Akt signaling compared to individual knockdowns. This provides valuable insights into downstream signal transducers associated with this signaling pathway. Importantly, the knockdown of gangliosides led to the inhibition of cell growth and migration through the FAK-paxillin-Akt signaling pathway, ultimately culminating in cellular apoptosis. These findings parallel the inhibitory effects observed in melanoma cells treated with quercetin.

In conclusion, our comprehensive evaluation supports quercetin’s potential anti-cancer efficacy against melanoma cells, positioning it as a promising candidate for targeted anti-cancer therapy. The observed role of ganglioside GD3, particularly in cellular growth and migration, highlights its significance as a potential therapeutic target for melanoma. Together, these findings contribute to the expanding body of evidence supporting quercetin as a potential anti-melanoma therapeutic agent and provide insights into the intricate interplay between quercetin, gangliosides, and signaling pathways in melanoma cells. Further research in this direction holds promise for advancing our understanding and refining therapeutic approaches for melanoma patients.

## 4. Materials and Methods

### 4.1. Reagent and Antibodies

Quercetin was acquired from Tokyo Chemical Industry (Tokyo, Japan) and was dissolved in dimethyl sulfoxide (DMSO; Sigma-Aldrich, St. Louis, MO, USA) to prepare the stock solution. All remaining reagents were procured from Sigma-Aldrich (St. Louis, MO, USA). The following antibodies obtained from Santa Cruz (Santa Cruz Biotechnology Inc., Santa Cruz, CA, USA) were utilized: anti-p53 (C-11), anti-cyclin D (M-20), anti-cyclin E (M-20), anti-cdk4 (C-22), anti-cdk6 (DSC-90), anti-Bax (P-19), anti-Bcl-xL (H-5), anti-caspase 3 (L-18), anti-cleaved caspase 3 p11 (h176), anti-caspase 8 p18 (D-8), anti-caspase 9 (96.1.23), anti-cleaved caspase 9 p10 (h331), anti-cytochrome c (A-8), anti-GM3 synthase (T-19), anti-GD3 synthase (B-11), and anti-β-actin (C-4). Additional antibodies (anti-Bid, anti-PARP, anti-MTCO1, and anti-tubulin β) were sourced from Cell Signaling Technology (Danvers, MA, USA) and Novus Biologicals (Novous Biologicals, LLC, Centennial, CO, USA).

### 4.2. Cell Culture

The melanoma cell lines (SK-MEL-28 and G-361) utilized in the current study were procured from the Korea Cell Line Bank (Seoul, Republic of Korea). These cells were cultured in Dulbecco’s Modified Eagle’s Medium (WelGene, Gyeongsan, Republic of Korea) supplemented with 10% fetal bovine serum (FBS; Gibco, Gaithersburg, MD, USA) and 100 U/mL penicillin-streptomycin (PS; Gibco, Gaithersburg, MD, USA) at 37 °C with 5% CO_2_ in a humidified incubator. Cell dissociation was performed using 0.25% trypsin-EDTA (Gibco, Gaithersburg, MD, USA).

A non-tumoral human epithelial cell line, HaCaT cells, was generously provided by Professor Jong Kun Park at Wonkwang University (Iksan, Republic of Korea). HaCaT cells were cultured in RPMI-1640 medium (WelGene, Gyeongsan, Republic of Korea) supplemented with 10% FBS and 100 U/mL PS at 37 °C and 5% CO_2_ in a humidified incubator until utilized.

### 4.3. MTT Cell Viability Assay

The impact of quercetin on the viability of melanoma cells was assessed using 3-(4,5-dimethylthiazol-2-yl)-2,5-diphenyltetrazolium bromide (MTT) from Sigma-Aldrich Biotechnology, St. Louis, MO, USA. SK-MEL-28, G-361, and HaCaT cells were seeded in 96-multiwell plates (SPL, Pocheon, Republic of Korea) at a density of 1 × 10^3^ cells per well for 24 h (hr) and then exposed to varying concentrations of quercetin (50, 100, 150, 200, 250, and 300 μM) at two different time points (24 and 48 h). Following the incubation, 200 μL of a mixed medium solution, composed of 20 μL of MTT solution (5 mg/mL) in 1 × phosphate-buffered saline (PBS; Biosesang, Seongnam, Republic of Korea) and 180 μL of complete medium, was added to each well for 3 h. The mixed medium was aspirated, and 200 μL of DMSO was then added to each well. Absorbance was measured using a BioTek Synergy HTX-Multi Microplate Multimode Reader (Agilent, Santa Clara, CA, USA) at a wavelength of 540 nm.

### 4.4. Nuclear Staining with DAPI

The assessment of nuclear condensation and fragmentation levels in melanoma cells was conducted using 4′,6-diamidino-2-phenylindole (DAPI) obtained from Thermo Fisher Scientific, Frederick, MD, USA. Briefly, SK-MEL-28, G-361, and HaCaT cells (1 × 10^4^ cells/well) were seeded on 18 mm coverslips (Marienfeld Corp., Lauda-Königshofen, Germany) in 12-well plates (SPL, Pocheon, Republic of Korea). Subsequently, the cells were treated with various concentrations of quercetin for 24 h, fixed with 4% paraformaldehyde (Sigma, St. Louis, MO, USA) in Dulbecco’s modified phosphate-buffered saline (DPBS; WelGene, Gyeongsan, Republic of Korea) at room temperature (RT) for 20 min (min). Finally, the cells were counter-stained with 2 μg/mL DAPI staining solution for 10 min at RT in the dark and analyzed using a fluorescence microscope (Carl Zeiss, Ulm, Germany).

### 4.5. Cell-Cycle Analysis

Melanoma cells (1.0 × 10^4^ cells/mL) were seeded into 6-well plates (SPL, Pocheon, Republic of Korea) and cultured for 24 h. Following exposure to varying concentrations of quercetin (0, 100, 200, and 300 μM) for 24 h, cells were harvested through trypsinization and washed twice with DPBS. The collected samples were fixed in 70% ethyl alcohol (Samchun, Gangnam, Republic of Korea) at –20 °C until staining. After fixation, the samples were washed with DPBS, recovered by centrifugation at 300× *g* for 5 min, and then treated with 200 μL of Muse^TM^ Cell Cycle Reagent (Luminex Corp., Austin, TX, USA). Following gentle suspension, the mixed samples were incubated at RT for 30 min in the dark. Cell analysis was ultimately performed by flow cytometry using a Muse^TM^ Cell Analyzer (Merck Millipore, Darmstadt, Germany).

### 4.6. Annexin V & Dead Cell Assay

SK-MEL-28 cells (2.0 × 10^5^ cells/mL) and G-361 cells (2.0 × 10^5^ cells/mL) were individually seeded on a 60 mm dish (SPL, Pocheon, Republic of Korea) and cultured for 24 h. Following treatment with varying concentrations (100, 200, and 300 μM) of quercetin for 24 h, cells were harvested through trypsinization and washed twice with DPBS. For the assessment of cell apoptosis, cells were stained using the Muse^TM^ Annexin V & Dead Cell kit (Luminex Corp., Austin, TX, USA) following the manufacturer’s instructions. Subsequently, 100 μL of Muse^TM^ Annexin V & Dead Cell reagent was added to each tube and incubated for 20 min at RT in the dark. The stained samples were then analyzed using a Muse^TM^ Cell Analyzer (Merck Millipore, Darmstadt, Germany).

### 4.7. Reverse Transcription-Polymerase Chain Reaction (RT-PCR) Analysis

Total RNA was isolated from melanoma cells (1.0 × 10^4^ cells/60 mm culture dish) using TRIzol^TM^ Reagent (Thermo Fisher Scientific, Frederick, MD, USA). Subsequently, 1.0 µg of RNA was utilized for cDNA synthesis through the AccuPower^®^ RT PreMix kit (Bioneer Corp., Daejeon, Republic of Korea). The cDNA for RT-PCR was generated by amplification with primers specific for β-actin, ST3 beta-galactoside alpha-2,3-sialyltransferase 5 (ST3GAL5), and ST8 alpha-N-acetyl-neuraminide alpha-2,8-sialyltransferase 1 (ST8SIA1). The RT-PCR was conducted using a TaKaRa PCR Thermal Cycler Dice Gradient System (Takara, Dalian, China). Detailed primer information and the specific PCR conditions employed in this study are provided in Table S1.

### 4.8. Mitochondrial Membrane Potential (MMP) Assay

The measurement of mitochondrial membrane potential (MMP) was conducted using the JC-1 MMP Assay kit (Abcam, Cambridge, UK) following the manufacturer’s instructions. Melanoma cells (1 × 10^5^ cells/well) were seeded on an 18 mm coverslip in 12-well plates and allowed to adhere. The cells were treated with various concentrations of quercetin (100 and 200 μM) for 24 h, washed twice with DPBS, and then 5 μM of JC-1 solution was added in 1 × dilution buffer. The samples were incubated at 37 °C for 15 min in the dark and subsequently washed twice with DPBS. The analysis of MMP in melanoma cells was performed using a fluorescence microscope (Carl Zeiss, Ulm, Germany).

### 4.9. Isolation of Mitochondrial and Cytosolic Protein Fractionation

Mitochondrial and cytosolic fractions were obtained using the Mitochondria and Cytosol Fractionation Kit (Abcam, Cambridge, UK) following the manufacturer’s protocols. After 24 h treatment with quercetin, melanoma cells were harvested and combined with 1 mL of 1 × cytosol extraction buffer containing DTT and protease inhibitors. Following 10 min incubation on ice, cells were homogenized using an ice-cold glass tissue grinder (Dounce, Wheaton, Millville, NJ, USA) for 30–50 passes. The samples were then centrifuged at 700× *g* at 4 °C for 10 min. The supernatants were transferred to new tubes and subjected to subsequent centrifugation at 13,000 rpm for 30 min to separate the mitochondrial fractions (pellets) and cytosolic fractions (supernatant). The mitochondrial pellets were washed with isolation buffer and lysed in mitochondrial extraction buffer containing DTT and protease inhibitors. All isolated samples were stored at −80 °C until further use.

### 4.10. Immunoblotting Analysis

Melanoma cell cultures, each comprising 1.0 × 10^6^ cells/mL, were subjected to various concentrations of quercetin (100, 200, and 300 µM) for distinct temporal intervals (12 and 24 h), followed by dual washes with DPBS. Cellular lysis ensued through the application of a protein extraction solution (RIPA) sourced from ELPIS Biotech Inc., Daejeon, Republic of Korea. Quantification of the extracted proteins was executed using the Pierce^®^ BCA Protein Assay kit (Thermo Fisher Scientific, Frederick, MD, USA).

Equal quantities of the protein samples underwent separation through sodium dodecyl sulfate-polyacrylamide gel electrophoresis at varying concentrations (6, 8, 10, or 15%), alongside a standard protein marker (Thermo Fisher Scientific, Frederick, MD, USA). Subsequent to electrophoresis, the proteins were transferred onto polyvinylidene fluoride membranes (Merck Millipore, Billerica, MA, USA). Membranes were subjected to a blocking step at RT for 1 h in a solution comprising 10 mL of 0.3% non-fat dried milk in 1 × Tris-buffered saline with 0.1% Tween-20 (Bio-Rad Inc., Hercules, CA, USA). Following this, the membranes were incubated overnight at 4 °C with specific primary antibodies (1:500–1:1000, *v*/*v*).

Subsequent to primary antibody incubation, the membranes underwent 2 h incubation at RT with horseradish peroxidase-conjugated anti-mouse, anti-rabbit, and anti-goat secondary antibodies (1:5000, *v*/*v*). Protein bands were visualized utilizing the Super Signal West Pico PLUS chemiluminescent substrate (Thermo Fisher Scientific, Frederick, MD, USA), and the resultant bands were imaged using a ChemiDoc Imaging system (Bio-Rad Laboratories, Inc., Hercules, CA, USA).

### 4.11. Immunocytochemistry Assay

Melanoma cells (1.0 × 10^6^ cells/mL) were cultured on aseptic coverslips within 12-well plates (SPL, Pocheon, Republic of Korea) for a 24 h duration, followed by exposure to quercetin for an additional 24 h. Cellular fixation involved a 30 min treatment with 4% paraformaldehyde (Sigma-Aldrich, St. Louis, MO, USA) at RT, succeeded by permeabilization using 0.1% Triton X-100 (Sigma-Aldrich, St. Louis, MO, USA) for 30 min at RT.

Blocking procedures were executed with DPBS containing 0.3% bovine serum albumin (Sigma-Aldrich, St. Louis, MO, USA) for 1 h at RT. Primary antibodies were applied at a 1:250 (*v*/*v*) dilution and incubated overnight at 4 °C. Subsequent to thorough washing with ice-cold PBS, cells underwent incubation with Alexa Fluor 546-conjugated (Invitrogen, Carlsbad, CA, USA) and Alexa Fluor 488-conjugated secondary antibodies (Invitrogen, Carlsbad, CA, USA) at a 1:500 (*v*/*v*) dilution at RT for 1 h. Nuclei were stained using 2 μg/mL DAPI reagent. Visualization of cells was achieved using a fluorescence microscope (Carl Zeiss, Ulm, Germany).

### 4.12. Wound Healing Assay

Melanoma cells (1 × 10^6^ cells/well) were plated in 60 mm culture dishes and allowed to adhere for a 24 h period. The creation of a wound involved a meticulous process; specifically, the central region of the monolayer was delicately scraped using a sterile pipette tip and subsequently washed twice with DPBS to eliminate cellular debris. Post-scratching, cells underwent treatment with 250 µM of quercetin and were cultured for 24, 48, and 72 h to facilitate the observation of cell migration dynamics under a Carl Zeiss microscope, based in Ulm, Germany.

### 4.13. Ganglioside Extraction and Purification

Gangliosides were prepared following established protocols as outlined in previous study [54]. Total lipids were extracted utilizing a chloroform/methanol solvent mixture (1:1, *v*/*v*). Subsequently, the separation of neutral lipids involved filtration with 20 mL of chloroform/methanol/H_2_O (15:30:4, *v*/*v*/*v*) through a DEAE Sephadex A25 column (Sigma-Aldrich, St. Louis, MO, USA), while acidic lipids were extracted using 15 mL of chloroform/methanol/0.8 M sodium acetate (15:30:4, *v*/*v*/*v*). Eluted samples were dried under N_2_ gas at 30 °C, dissolved in chloroform/methanol (1:1, *v*/*v*), and neutralized with 12 N NH_4_OH overnight at RT. Following a subsequent drying step with N_2_ gas at 30 °C, the neutralized samples were dissolved in distilled water, and salt removal was accomplished using a Sep-Pak C18 cartridge (Merk Millipore, Madison, WI, USA) to obtain total gangliosides. Ultimately, eluted gangliosides were dried under N_2_ gas at 30 °C for 4 h. These dried samples were then stored at −80 °C until further experimental procedures.

### 4.14. High-Performance Thin-Layer Chromatography (HPTLC)

HPTLC (High-Performance Thin-Layer Chromatography) analysis was conducted following established procedures as detailed in prior study [55]. Purified gangliosides dissolved in chloroform/methanol (1:1, *v*/*v*) were meticulously spotted using a capillary tube onto HPTLC plates measuring 10 × 10 cm (Merck Millipore, Darmstadt, Germany). Subsequent to spotting, the plates were developed using a solution comprising 100 mL of chloroform/methanol/0.25% CaCl_2_·H_2_O (50:40:10, *v*/*v*/*v*).

The developed gangliosides on the HPTLC plates underwent staining with a resorcinol solution (HCl, 0.1 M CuSO_4_·5H_2_O, resorcinol, distilled water), and the resultant samples were dried at 105 °C in a dry oven for a duration of 2 h. For reference, the monosialoganglioside mixture (Matreya LLC, State College, PA, USA) and disialoganglioside mixture (Matreya LLC, State College, PA, USA) served as standard markers to characterize individual ganglioside species.

### 4.15. Knockdown of Ganglioside ST3GAL5 and ST8SIA1 Gene by Small Interfering RNA (siRNA)

Melanoma cells were seeded at a density of 1.5 × 10^5^ cells/well in a 6-well plate (Thermo Fisher Scientific, Frederick, MD, USA) and cultured overnight. Ganglioside GM3 and GD3 synthase-specific siRNA, as well as control siRNA, were custom-synthesized by Thermo Fisher Scientific (Frederick, MD, USA). The primers designed for GM3 synthetase (ST8SIA1) were as follows: Forward, 5′-GUCAGUUAGUGACAGCUAAtt-3′, and Reverse, 5′-UUAGCUGUCACUAACUGACtt-3′. For GD3 synthetase (ST3GAL5), the primers were Forward, 5′-CGAUGUUGUGAUAAGGUUAtt-3′, and Reverse, 5′-UAACCUUAUCACAACAUCGaa-3′.

Cell transfections were performed using Opti-MEM medium (Invitrogen, Carlsbad, CA, USA) and Lipofectamin^®^3000 reagent (Invitrogen, Carlsbad, CA, USA) with ST8SIA1 siRNA or ST3GAL5 siRNAs (10 nM), following the manufacturer’s instructions. After a four-day incubation, the Opti-MEM medium was replaced with regular culture medium. The impact of downregulation on ganglioside synthase ST3GAL5 or ST8SIA1 was evaluated 48 h post-transfection through RT-PCR and immunoblotting assays, respectively.

### 4.16. Data Analysis

All data underwent statistical analysis for significance utilizing one-way ANOVA, followed by post hoc Tukey’s multiple range test, employing GraphPad Prism (Ver. 5.00; GraphPad Software Inc., La Jolla, CA, USA). Results are expressed as the mean ± standard deviation from three independent experiments. Statistical significance was established at *p* < 0.05.

## 5. Conclusions

In summary, our study highlights the potential of quercetin as a multifaceted therapeutic agent against melanoma. Through its ability to arrest the G0/G1 cell cycle, induce mitochondrial apoptosis, and decrease ganglioside GM3 and GD3, quercetin exerts negative regulation on Raf-Akt signaling, leading to the attenuation of melanoma cell proliferation and activation. Furthermore, our findings on the suppression of ganglioside GD3, which reduces cell growth and proliferation by negatively regulating FAK-paxillin-Akt signaling, reinforce the significance of gangliosides as potential therapeutic targets. These results recommend quercetin and ganglioside-targeted therapies for combating melanoma progression and offer a potential candidate for use as a therapeutic.

## Data Availability

The datasets used and/or analyzed during the current study are available from the corresponding author upon reasonable request.

## Supplementary Materials

The following supporting information can be downloaded at https://www.mdpi.com/article/10.3390/ijms25105146/s1.
