# Supplementary material for: Quercetin Induces Mitochondrial Apoptosis and Downregulates Ganglioside GD3 Expression in Melanoma Cells
Source: Int J Mol Sci. 2024 May 9;25(10):5146. doi: 10.3390/ijms25105146 (PMC11121576; doi:10.3390/ijms25105146)
Supplement: Supplementary file 1 [file ijms-25-05146-s001.zip › ijms-2983128-supplementary.pdf]

Quercetin Induces Mitochondrial Apoptosis and Downregulates Ganglioside GD3 Expression in Melanoma Cells

S ang Young Seo[1,2,†], Won Seok Ju[1,3,†], Kyongtae Kim[1], Juhwan Kim[1], Jin Ok Yu[1], Jae-Sung Ryu[4], Ji-Su Kim[5], Hyun-A Lee[6], Deog-Bon Koo[7] and Young-Kug Choo[1,8,*]

[1]Department of Biological Science, College of Natural Sciences, Wonkwang University, 460, Iksan-daero, Iksan-si, Jeollabuk-do 54538, Republic of Korea

[2]Division of Animal Diseases & Health, National Institute of Animal Science, Rural Development Administration, 1500 Kongjwipatjwi-ro, Iseo-myeon, Wanju-gun, Jeonbuk 55365, Republic of Korea

[3]Animal Biotechnology Division, National Institute of Animal Science, Rural Development Administration, 1500 Kongjwipatjwi-ro, Iseo-myeon, Wanju-gun, Jeonbuk 55365, Republic of Korea

[4]Division of drug evaluation, New drug development center, Osong medical innovation foundation, Cheonju-si, 28160, Republic of Korea

[5]Primate Resources Center (PRC), Korea Research Institute of Bioscience and Biotechnology (KRIBB), 181, Ipsin-gil, Jeongeup-si, Jeollabuk-do 56216, Republic of Korea

[6]Center for Animal Resources Development, Wonkwang University, 460, Iksan-daero, Iksan-si, Jeonbuk 54538, Republic of Korea

[7]Department of Biotechnology, College of Engineering, Daegu University, 201 Deagudae-ro, Jillyang, Gyeongsan, Gyeongbuk 38453, Republic of Korea.

[8]Institute for Glycoscience, Wonkwang University, 460, Iksan-daero, Iksan-si, Jeollabuk-do 54538, Republic of Korea

[†]These two authors contributed equally to this work

[*]Correspondence to: Young-Kug Choo, Department of Biological Science, College of Natural Sciences, Wonkwang University, 460, Iksan-daero, Iksan-si, Jeollabuk-do 54538, Republic of Korea, Tel.: +82-63-850-6087, Fax: +82-63-857-8837, E-mail: ykchoo@wku.ac.kr

Supplementary Figure Legends

Table S1. Primer sequences used to generate templates for revers transcription-polymerase chain reaction (RT-PCR).

Figure S1. Changes in ganglioside expression in quercetin-treated SK-MEL-28, G-361 and non-tumoral HaCaT cells. (A) RT-PCR and (B) western blot analysis of GM3 and GD3 synthase ezpression in SK-MEL-28 and G-361 cells. ACTB was used as a loading control. ACTB; β-actin. The results shown are representative of at least three independent experiments ($n = 3$, $^*p < 0.05$). (**C**) HPTLC analysis of gangliosides in untreated HaCaT cells (L1) and HaCaT cells treated with quercetin 250 μM for 24 h (L2). M, marker; L, line; M1 and M2, ganglioside standard mixture marker (left panel); the quantification of band intensity of ganglioside GM3, GM1, and GD3 in HaCaT cell lines (right panel). White and black squares indicate untreated HaCaT cells and HaCaT treated with quercetin cells, respectively. Data represent mean ± SE ($n = 3$, $^*p < 0.05$). (D) RT-PCR analysis and (E) western blot analysis of GM3 and GD3 synthase expression in non-tumoral HaCaT cells. ACTB was used as a loading control. ACTB; β-actin. The results shown are representative of at least three independent experiments ($n = 3$, $^*p < 0.05$).

Figure S1

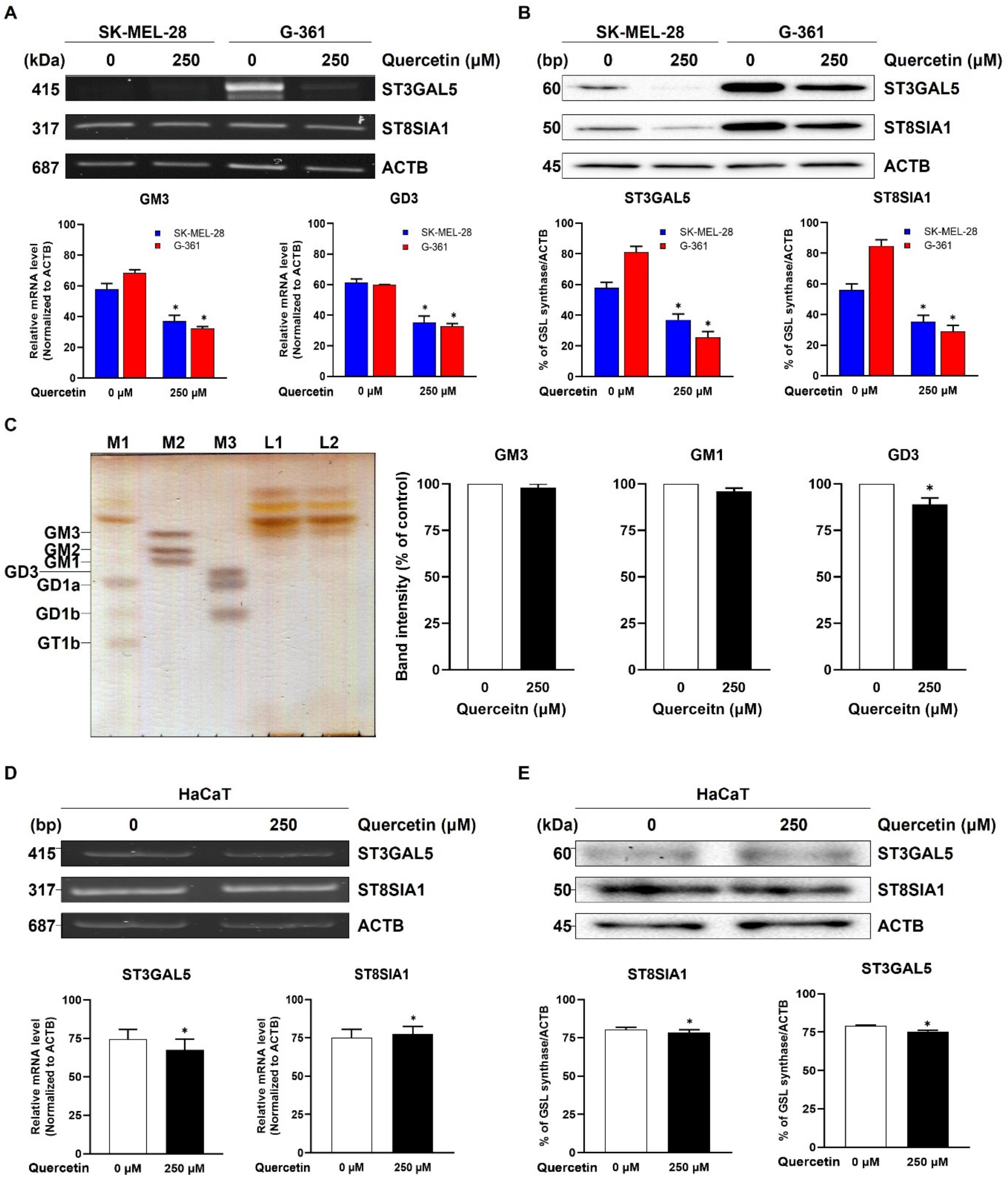

A
SK-MEL-28
G-361
(kDa)
0
250
Quercetin (μM)
415
ST3GAL5
317
ST8SIA1
687
ACTB
GM3
GD3
Relative mRNA level (Normalized to ACTB)
SK-MEL-28
G-361
Quercetin
0 μM
250 μM
B
(bp)
60
50
45
ST3GAL5
ST8SIA1
% of GSL synthase/ACTB
C
M1
M2
M3
L1
L2
GM3
GM2
GM1
GD3
GD1a
GD1b
GT1b
GM1
Band intensity (% of control)
Querceitn (μM)
D
HaCaT
E

Table S1

Primer sequences used to generate templates for RT-PCR.

Gene	Description	Species	Primer (5′→3′)	Reaction condition	Size (bp)
ST3GAL5	ST3 beta-galactoside alpha-2,3-sialyltransferase 5	*Homo sapiens*	F: CCC TGA ACC AGT TCG ATG TT<br>R: GTG GCT AAG ACA ACG GCA AT	Denaturation: 30 s at 95 °C<br>Annealing: 30 s at 62 °C<br>Extension: 30 s at 72 °C<br>Reaction cycles: 35	415
ST8SIA1	ST8 alpha-N-acetylneuraminide alpha-2,8-sialyltransferase 1	*Homo sapiens*	F: GCA ATC TCC CTC CTC CTT TGT CA<br>R: GCT TGG CAT GGA TTC CTC TA		321
ACTB	β-Actin	*Homo sapiens*	F: CGC AAG TAC TCC GTG TGG AT<br>R: GTC AGT GTA CAG GTA AGC CCT G		687
